# Astacin Proteases Cleave Dentin Sialophosphoprotein (Dspp) to Generate Dentin Phosphoprotein (Dpp)

**DOI:** 10.1002/jbmr.202

**Published:** 2010-08-04

**Authors:** Shuhei Tsuchiya, James P Simmer, Jan C-C Hu, Amelia S Richardson, Fumiko Yamakoshi, Yasuo Yamakoshi

**Affiliations:** 1 Department of Biologic and Materials Sciences, University of Michigan School of DentistryAnn Arbor, MI 48108, USA; 2 Department of Maxillofacial Surgery/Protective Care for Masticatory Disorders, Nagoya University Graduate School of MedicineNagoya, Japan

**Keywords:** DSPP, BMP-1, ASTACIN, PHOSPHOPHORYN, DENTIN, TEETH

## Abstract

Dentin sialophosphoprotein (Dspp) is critical for proper dentin biomineralization because genetic defects in *DSPP* cause dentin dysplasia type II and dentinogenesis imperfecta types II and III. Dspp is processed by proteases into smaller subunits; the initial cleavage releases dentin phosphoprotein (Dpp). We incubated fluorescence resonance energy transfer (FRET) peptides containing the amino acid context of the Dpp cleavage site (YEFDGKSMQGDDPN, designated Dspp-FRET) or a mutant version of that context (YEFDGKSIEGDDPN, designated mutDspp-FRET) with BMP-1, MEP1A, MEP1B, MMP-2, MMP-8, MMP-9, MT1-MMP, MT3-MMP, Klk4, MMP-20, plasmin, or porcine Dpp and characterized the peptide cleavage products. Only BMP-1, MEP1A, and MEP1B cleaved Dspp-FRET at the G–D peptide bond that releases Dpp from Dspp in vivo. We isolated Dspp proteoglycan from dentin power and incubated it with the three enzymes that cleaved Dspp-FRET at the G–D bond. In each case, the released Dpp domain was isolated, and its N-terminus was characterized by Edman degradation. BMP-1 and MEP1A both cleaved native Dspp at the correct site to generate Dpp, making both these enzymes prime candidates for the protease that cleaves Dspp in vivo. MEP1B was able to degrade Dpp when the Dpp was at sufficiently high concentration to deplete free calcium ion concentration. Immunohistochemistry of developing porcine molars demonstrated that astacins are expressed by odontoblasts, a result that is consistent with RT-PCR analyses. We conclude that during odontogenesis, astacins in the predentin matrix cleave Dspp before the DDPN sequence at the N-terminus of Dpp to release Dpp from the parent Dspp protein. © 2011 American Society for Bone and Mineral Research.

## Introduction

Type 1 collagen is the major extracellular matrix protein in tooth dentin comprising about 85% to 90% of total protein.(1) Collagen 1 is a triple helix that contains two α_1_ and one α_2_ polypeptides, which are expressed from *COL1A1* (17q21.31-q22) and *COL1A2* (7q22.1). Mutations in these genes cause osteogenesis imperfecta (OI), an autosomal dominant form of “brittle bone disease.” Dentin malformations are often associated with OI,(2,3) and in some cases, the dental malformations are the most apparent part of the phenotype.(4) OI with dentin malformations as a feature is also classified as type 1 dentinogenesis imperfecta (DGI-1).(5)

The major noncollagenous proteins in human dentin are expressed from the dentin sialophosphoprotein (*DSPP*) gene (4q21.3). Dspp is a secreted protein that is rapidly cleaved into three products: dentin sialoprotein (Dsp),(6,7) dentin glycoprotein (Dgp).(8) and dentin phosphoprotein (Dpp),(9–11) although Dgp has been characterized only in pig. Dsp is a highly *N*-glycosylated proteoglycan containing two glycan chains comprised mainly of chondroitin 6-sulfate.(12) Dpp is notable for its extremely acidic isoelectric point (p*I* 1.1).(13) A major feature of the Dpp protein is an extensive region of phosphorylated DSS repeats. The DSS repeat region varies greatly in length, which accounts for the significant size differences of Dpp among mammals. The numbers of amino acids in human,(14,15) mouse,(16) and porcine Dpp(17) are known to vary even among individuals of the same species. Porcine Dpp varies in length between 551 and 594 amino acids. DPP in the normal human population varies between 758 and 902 amino acids.(15,18,19) Analysis of the DPP region from 188 human chromosomes identified 20 separate slip-replication indel events (length polymorphisms) in 38 different *DSPP* haplotypes.(15)

Mutations in *DSPP* cause a range of autosomal dominant dental diseases, including dentin dysplasia type 2 (DD-II),(20) dentinogenesis imperfecta type 2 (DGI-II),(21) and dentinogenesis imperfecta type 3 (DGI-III).(22) To date, no other gene besides *DSPP* has been associated with isolated inherited dentin defects.(2,14) *Dspp* null mice display severe dentin malformations,(23) while transgenic expression of only the N-terminal domain (Dsp) in the null background partially rescues the dentin phenotype.(24) The volume of dentin but not the dentin mineral density is restored. These results demonstrate distinct roles for the N-terminal and C-terminal (Dpp) domains of Dspp in dentin mineralization and suggest that the proteolytic cleavage that separates these functional components is important for normal dentin biomineralization.

The initial proteolytic cleavage of Dspp is in a conserved amino acid context and releases Dpp, so Dpp proteins isolated from various mammals share the same N-terminal sequence: DDPN. The amino acid context of the cleavage that releases Dpp coincides with the known target preferences of bone morphogenetic protein 1 (BMP-1).(25) BMP-1 is a member of the astacin family of the metzincin superfamily of zinc proteases. There are 80 metzincin genes in humans,(26) including 6 astacin genes [*BMP1*, 8p21.3; tolloid-like 1 (*TLL1*), 4q32-q33; tolloid-like 2 (*TLL2*), 10q23-q24; meprin Aα (*MEP1A*), 6p12-p11; meprin Aβ, (*MEP1B*), 18q12.2-q12.3; and astacin-like (*ASTL*) 2q11.1).(27) *Bmp1* transcripts are alternatively spliced to generate two proteases: BMP-1 and tolloid (TLD).(28) BMP-1/tolloid-like proteinases process dentin matrix protein 1 (Dmp1) in bone, and these cleavages do not appear to occur in mouse embryos that are homozygous null for genes encoding three of the four mammalian BMP-1/tolloid-like proteinases.(29) The *Bmp1* null condition is perinatal lethal(30); the *Tll* null and *Bmp1*/*Tll* double-null conditions are embryonic lethal.(31) Dspp is expressed only in trace levels in bone,(32) and the null mice die prior to significant tooth development, so these models cannot be used to determine whether the BMP-1/tolloid-like proteinases are required to catalyze the cleavage that releases Dpp from Dspp in dentin.

While the astacin proteases appear to have the appropriate target specificity to catalyze the Dpp cleavage, there is no direct evidence that these enzymes are expressed by odontoblasts (the cells that secrete the matrix that mineralizes to form dentin). Procollagen is processed by BMP-1 to remove C-terminal propeptides,(33,34) so BMP-1 generally is assumed to be produced by odontoblasts. However, all four of the BMP-1/tolloid-like proteinases can process procollagen,(35) and it is not known which of these enzymes process procollagen in dentin formation. The Dpp cleavage also might be catalyzed by any of the many proteases that have been identified in dentin(36–38) or autocatalyzed by the Dpp domain itself.(39) In this study, we use biochemical approaches to identify proteases that are able to cleave porcine Dspp at the exact cite that releases Dpp from the parent protein.

## Methods and Materials

### Subjects

All experimental procedures involving the use of animals are in compliance with the guiding principles in the *Care and Use of Animals* and were reviewed and approved by the Institutional Animal Care and Use Program at the University of Michigan.

### Tissue preparation and immunohistochemical analysis

Immunohistochemical analyses were performed with a Vectastain ABC Kit (Vector Laboratories, Inc., Burlingame, CA, USA). Briefly, the porcine third molar tooth bud was surgically removed from 6-month-old pigs and fixed in 4% paraformaldehyde for 24 hours at 4°C. The specimen was embedded in paraffin after decalcification with Morse's solution (10% sodium citrate, 20% formic acid) for 2 weeks and then sectioned to 5-µm thickness. Paraffin sections were dewaxed with xylene and rehydrated with decreasing concentrations of ethanol. To inactivate endogenous peroxidase, the sections were treated with 1% v/v hydrogen peroxide in methanol. Nonspecific binding of immunoglobulin was blocked by incubation with 4% v/v rabbit serum for 30 minutes at room temperature. The sections were incubated with goat antihuman BMP-1/PCP antibody (R&D Systems, Inc., Minneapolis, MN, USA) and diluted 2 µg/mL in PBS for 24 hours at 4°C. The sections then were washed in PBS and incubated for 60 minutes at room temperature with antigoat IgG, horseradish peroxidase–linked, diluted 1:200 in PBS, and reacted with 0.02% w/v 3,3'-diaminobenzidine tetrahydrochloride (Sigma-Aldrich, St Louis, MO, USA) in PBS containing 0.005% v/v hydrogen peroxide. The slides were washed in running water and stained with hematoxylin before being mounted and examined by light microscopy.

### Preparation of dentin powder

Tooth germs of permanent second molars were surgically extracted with a hammer and chisel from the maxillae and mandibles of 22 six-month-old pigs within minutes of each animal's termination at the Michigan State University Meat Laboratory (East Lansing, MI, USA). Two maxillary and two mandibular second molars were obtained from each animal. The developmental stage of the molars was advanced in crown formation but prior to the onset of root formation. Soft tissue (enamel organ epithelium and dental pulp) was removed with forceps. As much enamel as possible was removed by scraping with a curette. The remaining hard tissue was reduced to “dentin powder” using a jaw crusher (Retsch Inc., Newtown, PA, USA).

### Extraction of proteins from dentin powder

Dentin powder (50 g) was suspended with 50 mM Tris-HCl/4 M guanidine buffer (pH 7.4) containing Protease Inhibitor Cocktail Set III [1 mM 4-(2-aminoethyl)benzenesulfonyl fluoride hydrochloride (AEBSF), 0.8 µM aprotinin, 50 µM bestatin, 15 µM E-64, 20 µM leupeptin, and 10 µM pepstatin (Calbiochem EMD Chemicals Inc., Gibbstown, NJ, USA)] and 1 mM 1,10-phenanthroline (Sigma-Aldrich) and was homogenized using a Polytron (Capitol Scientific, Inc., Austin, TX, USA) homogenizer for 30 seconds at half speed. Insoluble material was pelleted by centrifugation (15,900*g*) and extracted two more times with the same buffer. The guanidine-insoluble material (30 g) was dialyzed against 16 L of 0.5 M acetic acid (HAc) containing 5 mM benzamidine (Sigma-Aldrich), 1 mM phenylmethylsulphonyl fluoride (PMSF; Sigma-Aldrich), and 1 mM 1,10-phenanthroline. Each day the calcium concentration in the reservoir was measured using the Calcium Reagent Set (Pointe Scientific, Canton, MI, USA), and the HAc was replaced. After 5 days, the calcium ion concentration of the HAc reservoir fell below 0.2 mM, indicating that the tooth mineral had fully dissolved. The dialysis bag contents were centrifuged. The supernatant of acid-soluble material (A extract) was lyophilized. The pellet was extracted with 0.5 M HAc/2 M NaCl. The supernatant (AN extract) was dialyzed against water, lyophilized, and used to isolate DPP. The AN pellet was extracted with 50 mM Tris/2 M NaCl. The supernatant (TN extract) was dialyzed against water, lyophilized, and used to isolate BMP-1-like protease.

Twenty grams of the guanidine-insoluble material was dialyzed against 16 L of 0.17 N HCl and 0.95% formic acid (HF) containing 10 mM benzamidine (Sigma-Aldrich), 1 mM PMSF, and 1 mM 1,10-phenanthroline for 1 day. Following centrifugation of the dialysis bag contents, the acid-soluble supernatant (HF-A extract) was lyophilized. The pellet was extracted with 0.5 M HAc/2 M NaCl and centrifuged, and the supernatant (HF-AN extract) was dialyzed against water, lyophilized, and used to isolate Dspp.

### Purification of porcine dentin Dpp

AN extract (∼10 mg) was dissolved in 0.05% trifluoroacetic acid (TFA) and fractionated by reversed-phase high-performance liquid chromatography (RP-HPLC) using a Discovery C-18 column (1.0 × 25 cm; Sigma-Aldrich/Supelco) run at a flow rate of 1.0 mL/minute and monitored at 220 nm (buffer A: 0.05% TFA; buffer B: 80% acetonitrile/buffer A). Dpp was found in the second of five fractions (AN-R2).

### Isolation of porcine dentin BMP-1-like protease

TN extract (∼100 mg) was fractionated by anion-exchange chromatography using a Q-Sepharose Fast Flow column (1.6 × 20 cm; GE Healthcare) equilibrated with buffer A: 50 mM Tris-HCl and 6 M urea (pH 7.4). Proteins were eluted with buffer A for 13 hours, a linear gradient of buffer B (buffer A + 0.5 M NaCl) for 13 hours, and a linear gradient of buffer C (buffer A + 2 M NaCl) for 6 hours at a flow rate of 0.2 mL/minute at 4°C while monitoring the absorbance at 280 nm. The BMP-1-immunopositive fraction, which was eluted in the third peak (TN-Q3), was dialyzed against water, lyophilized, and stored at −80°C.

### Isolation of porcine dentin sialophosphoprotein (Dspp)

HF-AN extract (120 mg) was fractionated by anion-exchange chromatography using a Q-Sepharose Fast Flow column (1.6 × 20 cm; GE Healthcare) equilibrated with buffer A: 50 mM Tris-HCl and 6 M urea (pH 7.4). Proteins were eluted with buffer A for 7 hours, a linear gradient of buffer B (buffer A + 0.2 M NaCl) for 10 hours, a linear gradient of buffer C (buffer A + 0.5 M NaCl) for 10 hours, and a linear gradient of buffer D (buffer A + 2 M NaCl) for 7 hours at a flow rate of 0.2 mL/minute at 4°C while monitoring the absorbance at 280 nm. Dspp eluted in the third peak (HF-ANQ3) was dialyzed against water, lyophilized, and stored at −80°C. The HF-ANQ3 extract (70 mg) was dissolved in 0.05% ammonium hydroxide (NH_4_ OH) and fractionated by RP-HPLC using a POROS R2/10 column (4.6 mm × 10 cm, Applied Biosystems/Life Technologies Corp., Carlsbad, CA, USA) at a flow rate of 1.0 mL/minute and monitored at 220 nm (buffer A: 0.05% TFA; buffer B: 80% acetonitrile/buffer A). Dspp eluted in the second peak. The sample was rechromatographed twice under the same conditions.

### rhBMP-1, rhMEP1A, or rhMEP1B digestion of porcine Dspp for HPLC

Purified Dspp (500 µg each) was dissolved with 500 µL of 25 mM HEPES, 2 mM CaCl_2_, 1 mM ZnCl_2_, 0.01% Brij 35, and 0.5 mM AEBSF buffer (pH 7.5) and was digested with rhBMP-1, rhMEP1A or rhMEP1B (2 µg each) at 37°C, respectively. Reaction aliquots at 0, 0.5, 1, 2, 5, 10, and 20 hours were analyzed by SDS-PAGE, and the digestion products at 20 hour were characterized by RP-HPLC using a Discovery C-18 column (4.6 × 25 cm; Sigma-Aldrich/Supelco) run at a flow rate of 0.8 mL/min and monitored at 220 nm (buffer A: 0.05% TFA; buffer B: 80% acetonitrile/buffer A). The N-terminus of the generated Dpp was determined by Edman degradation.

### Digestion of Dspp-FRET and mutDspp-FRET peptides by proteinases

Two fluorescence resonance-energy-transfer (FRET) peptides designated Dspp-FRET (Abz-YEFDGKSMQGDDPN-KDnp) and its mutated peptide designated mutDspp-FRET (Abz-YEFDGKSIEGDDPN-KDnp) were synthesized and purified by JPT Peptide Technologies (Berlin, Germany). The peptides were labeled with 2-aminobenzoyl (Abz) and 2,4-dinitrophenyl (Dnp). Recombinant human BMP-1 (rhBMP-1), MMP-2 (rhMMP-2), MMP-8 (rhMMP-8), MMP-9 (rhMMP-9), meprin α subunit (rhMEP1A), meprin β subunit (rhMEP1B), MT1-MMP (rhMT1-MMP), and MT3-MMP (rhMT3-MMP) were purchased from R&D Systems. Three MMPs (rhMMP-2, rhMMP-8, and rhMMP-9; 10 µg each) were activated with 1 mM *p*-aminophenylmercuric acetate in 50 mM Tris-HCl/10 mM CaCl_2_ /150 mM NaCl/0.05% Brij35 buffer (pH 7.5; TCNB buffer) at 37°C for 1 hour against rhMMP-2 and rhMMP-8 and for 24 hours against rhMMP-9. Two meprins (rhMEP1A and rhMEP1B; 10 µg each) were activated with 0.1 µg of trypsin (Sigma-Aldrich) in TCNB buffer at 37°C for 3 hours against rhMEP1A and for 45 minutes against rhMEP1B. The activation was stopped by adding AEBSF (R&D Systems) at a final concentration of 1 mM in TCNB buffer. The rhMT1-MMP and rhMT3-MMP (10 µg each) were activated with 156 ng of furin (R&D Systems) in 50 mM Tris-HCl/1 mM CaCl_2_ buffer (pH 8.8) at 37°C for 90 minutes. Porcine Mmp-20 and Klk4 were prepared as described previously.(40) Proteinase activities were detected on gelatin or casein gel by zymography.

Dspp-FRET and mutDspp-FRET (30 µg each) were incubated with rhBMP-1 (1 µg), rhMMP-2 (1 µg), rhMMP-8 (1 µg), rhMMP-9 (1 µg), rhMT1-MMP (2.5 µg), rhMT3-MMP (2.5 µg), porcine Klk4 (10 µg), porcine MMP-20 (10 µg), or 0.1 unit of human placenta plasmin (Sigma-Aldrich) in 25 mM HEPES, 2 mM CaCl_2_, 1 mM ZnCl_2_, and 0.01% Brij 35 buffer (pH 7.5) at 37°C for 2 hours (rhBMP1, rhMEP1A, and rhMEP1B) or 24 hours (rhMMP-2, rhMMP-8, rhMMP-9, MMP-20, rhMT1-MMP, rhMT3-MMP, Klk4, and plasmin). Dspp-FRET also was incubated with 100 µg of porcine BMP-1-like protease or 100 µg of porcine Dpp at 37°C for 48 hours, as described earlier. Aliquots were analyzed by LCMS/MS or fractionated by RP-HPLC for N-terminal sequencing. The RP-HPLC elution was monitored using ultraviolet (220 nm) and fluorescence detectors (excitation λ 320 nm; emission λ 420 nm). To determine their activities, rhMEP1B was used to digest the commercial fluorogenic peptide (MCa-SEVNLDAEFR-KDnpRR); rhBMP-1 and rhMEP1A were used to digest Mca-YVADAP-KDnp-OH (R&D Systems).

### Digestion of Dpp by proteinases

Purified Dpp (30 µg) was incubated for 20 hours in 25 mM HEPES, 2 mM CaCl_2_, 1 mM ZnCl_2_, and 0.01% Brij 35 buffer (pH 7.5) at 37°C with 0.5 µg of rhMMP-2, rhMMP-8, rhMMP-9, rhMT1-MMP, rhMT3-MMP, rhBMP-1, rhMEP1A, or rhMEP1B, with 10 µg of porcine Klk4 or porcine MMP-20, or with 0.1 unit of human placental plasmin.

Two different concentrations of Dpp (1.5 mg/1.5 mL or 1.5 mg/0.5 mL) were incubated with 0.5 µg of rhMEP1B in HEPES buffer (described earlier) at 37°C for 20 hours, respectively. At the end of the digestion, each aliquot of the digest (3 to 5 µg) was analyzed by SDS-PAGE. To determine the calcium ion concentration, the digests were passed through a Microcon 3 filter (cutoff = 3,500 Da; Millipore, Billerica, MA, USA). The free calcium in the filtrate was analyzed using a calcium reagent set (Pointe Scientific). Twenty microliters of filtrate was added in 1 mL of 0.11 mM *o*-cresolphthalein complexone (OCPC)/17 mM 8-hydroxyquinoline/976 mM 2-amino-2-methyl-1-propanol/2 mM potassium cyanide solution. The intensity of the Ca-OCPC complex was detected at 570 nm. An aqueous calcium solution (10 mg/dL = 2.5 mM) was used for the standard. Calcium concentration was calculated by the formula: mmoles of calcium = absorbance of sample/absorbance of standard × concentration of standard (2.5 mM).

### SDS-polyacrylamide gel electrophoresis (SDS-PAGE)

SDS-PAGE was performed using Novex 12% or 4% to 20% Tris-glycine or NuPAGE 3% to 8% Tris-acetate, or NuPAGE 4% to 12% Bis-Tris gels (Invitrogen, Carlsbad, CA, USA). Samples were dissolved in Laemmli sample buffer (Bio-Rad Laboratories, Hercules, CA, USA), and electrophoresis was carried out using a current of 30 mA for 65 minutes (Tris-glycine gel) or 150 V for 1 hour (Tris-acetate gel) or 35 minutes (Bis-Tris gel), respectively. The gels were stained with Simply Blue Safe Stain (Invitrogen) or Stains-All (Sigma-Aldrich). The apparent molecular weights of protein bands were estimated by comparison with SeeBlue Plus2 Pre-Stained Standard (Invitrogen).

### Automated Edman degradation

Automated Edman degradation used the Applied Biosystems Procise 494 cLC protein sequencer at the W. M. Keck Facility at Yale University.

### Mass spectrometry (LC-MS/MS)

LC-MSMS was performed by Nextgen Sciences (Ann Arbor, MI, USA) on the digests of the Dspp-FRET peptide after 2 or 24 hours. Samples were analyzed by nano-LC/MS/MS on a ThermoFisher LTQ Orbitrap XL (Thermo Fisher Scientific Inc., Waltham, MA, USA).

### Enzymograms

Zymography was carried out using 10% Zymogram Gelatin Gel and 12% Zymogram Casein Gel (Invitrogen). Samples were dissolved in Laemmli sample buffer (Bio-Rad), and electrophoresis was carried out using a current of 30 mA for about 1 hour. The gel was shaken gently in 2.5% Triton X-100 solution for 1 hour at room temperature with one buffer change and then incubated for overnight with 10 mM CaCl_2_ in 50 mM Tris-HCl buffer (pH 7.4) at 37°C. Proteinase activities were visualized as unstained bands after the gel was stained with Coomassie Brilliant Blue (CBB) or double stained with CBB and Stains-All.

### Western blot analyses

Samples fractionated by SDS-PAGE were electrotransferred onto a Hybond-ECL membrane (GE Healthcare). The human BMP-1/PCP affinity-purified polyclonal antibody (R&D Systems) and the porcine Klk4 affinity-purified polyclonal antibody were used at dilutions of 1:200 and 1:100, respectively, and incubated overnight. The secondary antibody was diluted 1:10,000 and incubated for 3 hours. Immunopositive bands were visualized by chemiluminescent detection using the ECL Advance Western Blotting Detection Kit (Amersham Pharmacia, GE Healthcare Bio-Sciences Corp., Piscataway, NJ, USA).

### Reverse-transcriptase polymerase chain reaction (RT-PCR)

Isolation of odontoblast RNA from developing pig teeth was described previously.(41) Developing second molars in the crown-formation stage were surgically extracted from 5-month-old commercial pigs, and the enamel organ epithelial (EOE) and pulp were removed with tissue forceps. The hard tissue was rinsed with cold diethylpyrocarbonate (DEPC) water to inactivate RNases. Each pulp cavity was filled with 200 µL of lysis buffer (RNeasy Mini Kit, Qiagen, Valencia, CA, USA) to dissolve odontoblasts adhering to dentin. After micropipetting up and down several times, the buffer was transferred to an Eppendorf tube and frozen on dry ice. The odontoblast lysate was processed for total RNA isolation using the RNeasy Mini Kit and protocol for animal tissues (Qiagen). Purified total RNA was reverse transcribed using the SuperScript First-Strand Synthesis System for RT-PCR (Invitrogen). PCR amplifications used SuperMix Reagent (Invitrogen) and a melting temperature of 59°C and ran for 35 and 40 amplification cycles. Reaction mixes lacking template were amplified simultaneously as negative controls. The porcine-specific primer sets and expected product sizes were *Gapdh* (308 bp), forward: AAGTGGACATTGTCGCCATC, reversed: TCACAAACATGGGGGCATC; *Dmp1* (261 bp), forward: CCAGTGAGGAGAGCTTGGAC, reversed: TTGATTTGCTGCTGTCTTGG; *Dspp* (293 bp), forward: CTGTGGTCCCGGAAGATAGA, reversed: ATTCCCCGTTTCTTCACCTT; *Bmp1* (278 bp), forward: ATGAGACACTGGGAGAAGCA, reversed: TACTCCTGACCTGGCTGGAT; *Mep1A* (302 bp), forward: GGTGATCCCCAGAACTCAAA, reversed: ATCACCTGCCTGTTTTCCAC; *Mep1B* (350 bp), forward: GTTACAGTGGCCGTGTCCTT, reversed: CACTTGAGGTTGGGACTGGT.

## Results

### Identifying enzymes that cleave Dspp-FRET and Dpp

**Fig. 1:**
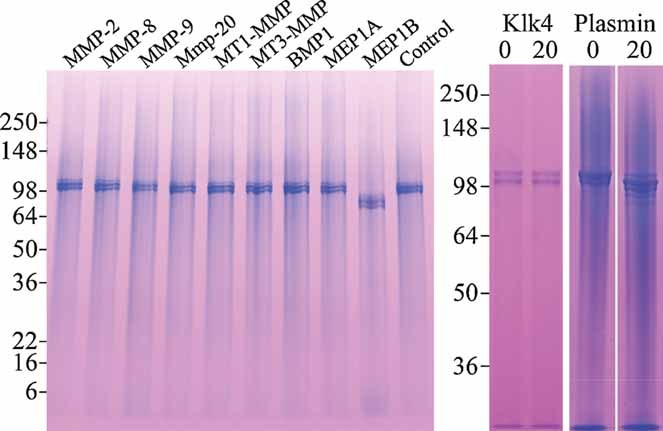
Digestion of porcine Dpp. Porcine Dpp migrates as two or three Stains-All^+^ bands at approximately 98 kDa. Porcine Dpp was not cleaved during a 20-hour incubation with rhMMP-2, rhMMP-8, rhMMP-9, porcine MMP-20, rhMT1-MMP, rhMT1-MMP, rhBMP-1, rhMEP1A, and porcine Klk4 and therefore comigrated with Dpp with no enzyme added (control). Human placental plasmin and rhMEP1B both cleaved Dpp to yield a faster-migrating protein.

**Fig. 2:**
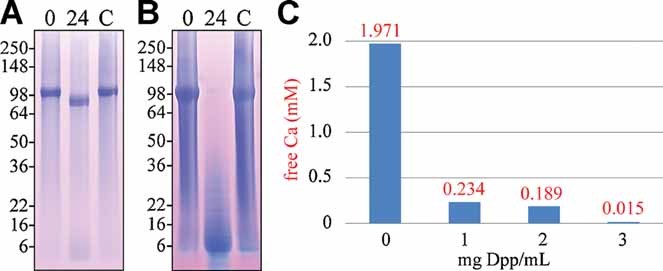
Digestion of Dpp by rhMEP1B. (*A*, *B*) SDS-PAGE (4% to 20% Tris/glycine gels) stained with Stains-All. Lanes 0: Digestion at *t* = 0. Lane 24: Digestion after 24 hours. Lane C: Controls with no enzyme added. (*A*) Digestion of porcine Dpp (1 mg/mL) that was added to a solution containing 2 mM CaCl_2_ and rhMEP1B. (*B*) Digestion of porcine Dpp (3 mg/mL) that was added to a solution containing 2 mM CaCl_2_ and rhMEP1B. (*C*) Graph of free calcium concentration of solutions containing 2 mM CaCl_2_ after no addition of Dpp (0) or after having added Dpp to a final concentration of 1, 2, or 3 mg/mL. The measured free calcium concentration are shown in red.

A panel of 11 proteases (rhMMP-2, rhMMP-8, rhMMP-9, MMP-20, rhMT1-MMP, rhMT3-MMP, Klk4, plasmin, rhBMP-1, rhMEP1A, and rhMEP1B) that are potentially associated with dentin formation were visualized on gelatin (Supplemental Fig. S1*A*) and casein (Supplemental Fig. S1*B*) zymograms. The rhBMP-1, rhMEP1A, and rhMEP1B were not visible on either zymogram; their activities were verified using commercially available FRET peptides containing appropriate target sequences (Supplemental Fig. S1*C–E*). The active enzymes or Dpp were incubated with FRET peptides containing the primary sequence context of the cleavage site that releases Dpp from Dspp (Dspp-FRET) or a “mutated” FRET peptide (mutDspp-FRET) that differed from the wild-type sequence at two amino acid positions. The FRET peptides in the absence of added protease were stable for at least 48 hours under reaction conditions. Porcine Dpp did not cleave Dspp-FRET (Supplemental Fig. S1*F*). The FRET peptides were incubated with or without added proteases, and the reaction products were separated by RP-HPLC and visualized using ultraviolet (UV) and fluorescence detectors (Supplemental Figs. S2 and S3). The positions of cleavages in the digested peptides were determined by Edman sequencing and by liquid chromatography–mass spectrometry (LC-MS/MS) and are shown in Table 1. Although eight of the proteases digested Dspp-FRET, only rhBMP-1, rhMEP1A, and rhMEP1B cleaved the peptide at the exact site (the G–D peptide bond) that releases Dpp from Dspp in vivo. The rhMMP-2, rhMMP-8, and rhMMP-9 cleaved the S–M peptide bond; Klk4 and plasmin both cleaved the K–S bond. Among the 11 enzymes, only rhMEP1B and plasmin could cleave Dpp at additional sites, causing it to migrate below the full-length Dpp domain on SDS-PAGE (Fig. 1). Under standard reaction conditions (1 mg/mL of Dpp/0.1 mg/mL of rhMEP1B in 2 mM CaCl_2_ ), rhMEP1B cleaved Dpp (∼98 kDa) to generate a modestly smaller product (∼85 kDa). When the Dpp concentration was raised to 3 mg/mL, which depleted the free calcium concentration, rhMEP1B degraded Dpp (Fig. 2).

**Table 1:** The 11 Enzymes and Dpp Used to Digest the FRET Peptides

Enzyme	Dspp-FRET	MutDspp-FRET
MMP-2	MQGDDPN	-
MMP-8	MQGDDPN	-
MMP-9	MQGDDPN	-
Mmp-20	-	-
MT1-MMP	-	-
MT3-MMP	-	-
Klk-4	SMQGDDPN	SIEGDDPN
Plasmin	SMQGDDPN	SIEGDDPN
BMP1	DDPN	-
MEP1A	DDPN	-
MEP1B	DDPN	DDPN

The amino acid sequences of the C-terminal cleavage products of Dspp-FRET (Abz-YEFDGKSMQGDDPN-KDnp) and mutDspp-FRET (Abz-YEFDGKSIEGDDPN-KDnp) were obtained by collecting the UV peaks not associated with a fluorescent peak and characterizing them by Edman degradation. A dash indicates that only the uncleaved peptide was identified at the end of the incubation.

### Isolation of Dspp

**Fig. 3:**
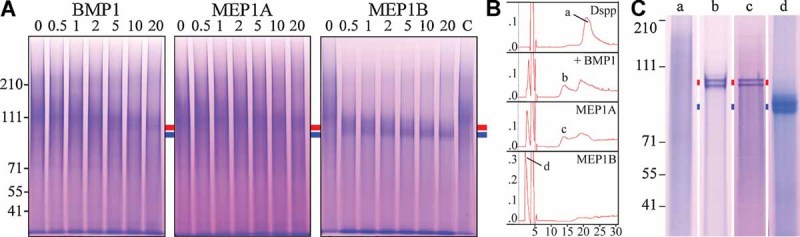
Digestion of porcine Dspp with rhBMP-1, rhMEP1A, and rhMEP1B. (*A*) SDS-PAGE (3% to 8% Tris/acetate gel) showing the digestions of the porcine Dspp proteoglycan by rhBMP-1, rhMEP1A, and rhMEP1B after 0, 0.5, 1, 2, 5, 10, and 20 hours or with no enzyme added (lane C). Dpp bands generated by rhBMP-1 and rhMEP1A during the digestion migrate at the red bar. Smaller Dpp bands generated by rhMEP1B migrate at the blue bar. (*B*) C-18 RP-HPLC chromatograms showing Dspp starting material (*a*) with a retention time between 20 and 25 minutes; the generation of peaks (*b*, *c*) with retention times of 14 minutes after digestion with rhBMP-1 and rhMEP1A; the generation of a peak at 3 minutes (*d*) after digestion with rhMEP1B. (*C*) SDS-PAGE (3% to 8% Tris/acetate gel) stained with Stains-All showing the contents of peaks *a* through *d* from panel *B*.

After multiple unsuccessful attempts to isolate intact (uncleaved) Dspp from porcine dentin, we changed the demineralization step from 0.5 M acetic acid for 6 days to 0.17 N HCl and 0.95% formic acid for 1 day. Although both methods included protease inhibitors, the shorter extraction under highly acidic conditions reduced sample degradation and enabled us to isolate intact Dspp. The purified Dspp displayed a single chromatographic peak on RP-HPLC with a retention time of between 20 and 25 minutes. Dspp fractionated by RP-HPLC and by SDS-PAGE showed no chromatographic peak with the retention time of Dpp (∼14 min) and no Stains-All^+^ Dpp bands migrating at 98 kDa on SDS-PAGE (Fig. 3). Thus the Dpp cleavage product was not present in the Dspp fraction. Dpp bands were generated from Dspp by digestion with rhBMP-1 and rhMEP1A (Fig. 3) and by these enzymes with serine protease inhibitors, but not when inhibited by EDTA (data not shown). Dpp bands were not generated in control incubations that lacked rhBMP-1 and rhMEP1A. Digestion of Dspp by rhBMP-1 and rhMEP1A generated a definite chromatographic peak at 14 minutes and characteristic Dpp bands migrating at 98 kDa on SDS-PAGE. Edman sequencing of the Dpp domain released from Dspp by digestion with rhBMP-1 or rhMEP1A gave the N-terminal sequence DDPNxxEE. In contrast, the smaller Dpp bands generated by the digestion of Dspp with rhMEP1B had a shorter retention time and gave the N-terminal sequence DDNGxD. The rhMEP1B cleavage removed an additional 36 amino acids from the N-terminus of Dpp.

### Astacin family proteases are present in porcine dentin

**Fig. 4:**
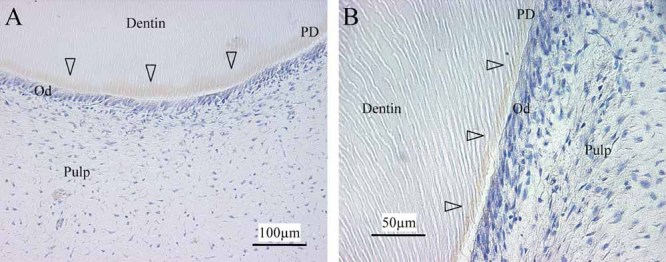
Localization of astacins (BMP-1-like proteases) in odontoblasts and predentin by immunohistochemistry. (*A*, *B*) Decalcified sections of developing third molars from a 6-month-old pig showing positive immunostaining (*arrowheads*) of odontoblasts and in predentin using a commercial antibody raised against rhBMP-1. Od = odontoblasts; PD = predentin.

**Fig. 5:**
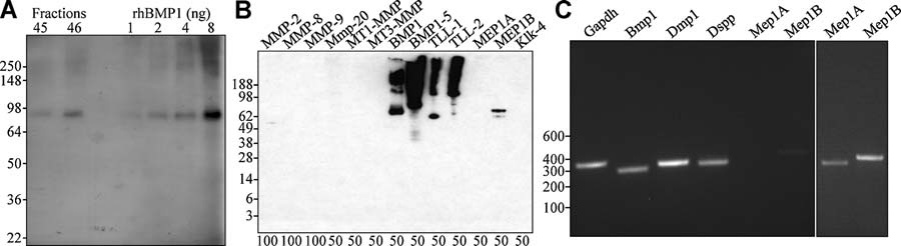
Analyses of dentin protease expression. (*A*) Western blot analysis using the commercial antibody raised against rhBMP-1 of anion-exchange samples 45 and 46 and increasing amounts of commercially available rhBMP-1 protein. (*B*) Western blot analysis using the commercial antibody raised against rhBMP-1 of enzymes used in this study. Below each lane is the amount of protein (ng) added to each lane. Note that rhBMP-1, rhBMP-1-5 (alternatively spliced isoform of hBMP-1), TLL-1, TLL-2, and MEP1B gave positive signal using the “BMP-1” antibody. This result suggests that this antibody recognizes the members of the astacin family. (*C*) Ethidium bromide–stained agarose gel showing RT-PCR products from porcine cells adhering to dentin after removal of the pulp. The gel on the left shows products after 35 cycles; on the right, after 40 cycles.

Since rhBMP-1 was able to cleave Dspp-FRET on the N-terminal side of the DDPN sequence, we sought additional evidence that BMP-1 is synthesized and secreted by odontoblasts and therefore is coexpressed with Dspp and available to cleave it in vivo. Developing porcine third molars tooth bud sections were immunostained using an antibody raised against BMP-1. Specific signal was detected in odontoblasts and in predentin (Fig. 4), but not in mineralized dentin. Next, we serially extracted porcine dentin powder to generate eight fractions. Each extract was characterized by gelatin and casein zymography and incubated with Dspp-FRET to determine which of the dentin powder extracts contained enzymes that could hydrolyze it (Supplemental Fig. S4). Fractions G1S and G1P hydrolyzed Dspp-FRET, but these fractions contain Klk4, which cleaves Dspp-FRET, but not at the exact site used to release Dpp from Dspp. The TN extract (but not extracts A, AN, and R) hydrolyzed Dspp-FRET. We used anion-exchange chromatography to separate the TN extract into six fractions (Supplemental Fig. S5). These fractions were characterized by SDS-PAGE stained with CBB and Stains-All and by Western blotting using the antibody raised against BMP-1 as a probe and using rhBMP-1 as a size and positive control. An immunopositive band was identified in fractions 45 and 46 from the anion-exchange separation that migrated at approximately 90 kDa on SDS-PAGE (Fig. 5*A*) and cleaved Dspp-FRET before the DDPN sequence corresponding to the N-terminus of Dpp isolated from dentin extracts. The immunopositive band comigrated with rhBMP-1 on SDS-PAGE (Fig. 5*A*). However, Western blot analysis showed that the commercial antibody raised against BMP-1 also recognized multiple members of the astacin family of enzymes, such as rhBMP-1, tolloid-like 1, tolloid-like 2, and MEP1B (Fig. 5*B*). RT-PCR of RNA isolated from cells clinging to coronal dentin following removal of the pulp (principally odontoblasts) was positive for the dentin matrix proteins Dspp and Dmp1, as well as BMP-1, Mep1A, and Mep1B (Fig. 5*C*). Taken together, our results demonstrate that member(s) of the astacin family of proteases are expressed and secreted by porcine odontoblasts and are uniquely capable of cleaving the G–D peptide bond in the primary-sequence context of the site that releases Dpp from Dspp in vivo.

## Discussion

The cleavages that divide Dspp into its component domains (Dsp, Dgp, and Dpp) are important because they allow these segments of the protein to function independently and diffuse to different locations in the matrix. Cleavage of the G–D bond that releases Dpp is the first cleavage of Dspp. In the pig, a subsequent cleavage by MMP-20 releases the 81-amino-acid Dgp glycoprotein from the C-terminal end of Dsp.(8,42) In the rat, there are at least two cleavages near the Dsp C-terminus, but the released peptides are not suspected of having a function, and the enzyme that catalyzes them has not been identified.(43) Recent attention has focused on the initial cleavage that releases Dpp. Gelatin zymogram evidence was used to conclude that the Dpp domain itself is a protease that catalyzes the self-processing of Dspp to releases Dpp.(39) However, Dpp shares no homology with any known protease, and its appearance as an unstained band on zymograms can be explained by the fact that Dpp doesn't stain with Coomassie brilliant blue.(42) Our finding that porcine Dpp does not cleave Dspp-FRET suggests that Dpp is not a protease and that further investigations are needed to characterize the mechanisms of Dspp processing.

While this article was under review, two studies were published implicating BMP-1 in the cleavage that releases Dpp from Dspp. Both studies showed that BMP-1 could cleave Dspp but could not cleave recombinant Dspp that had been mutated at or near the Dpp cleavage site.(44,45) Although there can be little doubt about the site cleaved, given the known target specificity of BMP-1, the position of the cleavage in Dspp was not determined experimentally. Instead, failure to cleave Dspp mutated near the known Dpp cleavage site was used to deduce that BMP-1 cleaves wild-type Dspp specifically at the Dpp site. Here we tested a panel of proteolytic enzymes associated with the dentin extracellular matrix for their ability to cleave Dspp-FRET and a mutated Dspp-FRET peptide. The mutDspp-FRET corresponded to the mutated Dspp sequence used in one of these studies.(45) Eight of the 11 enzymes were able to cleave Dspp-FRET, although only the 3 astacins (rhBMP-1, rhMEP1A, and rhMEP1B) were able to cleave the G–D bond that is cleaved in vivo to separate Dpp from Dspp. Three of the enzymes (rhMMP-2, rhMMP-8, and rhMMP-9) cleaved Dspp-FRET at a different site but could not cleave mutated Dspp-FRET. Furthermore, rhMEP1B was able to cleave both Dspp-FRET *and* mutDspp-FRET. These findings demonstrate that whether or not an enzyme such as BMP-1 specifically cleaves the G–D bond that releases Dpp from Dspp cannot be reliably inferred by mutating the cleavage-site context in the protein.

We isolated the Dspp proteoglycan from dentin power and incubated it with the three enzymes that cleaved Dspp-FRET at the G–D bond. In each case, the released Dpp domain was isolated and its N-terminus characterized by Edman degradation. rhBMP-1 and rhMEP1A both cleaved native Dspp at the G–D bond to generate Dpp, making both these enzymes prime candidates for the protease that cleaves Dspp in vivo. rhMEP1B generated a smaller Dpp product that was 36 amino acids shorter than native Dpp at its N-terminus. Furthermore, we discovered that rhMEP1B degrades Dpp in vitro when the Dpp concentration is raised to the point where free calcium is depleted. Some Dpp is apparently degraded in vivo as a natural part dentin formation. In porcine dentin extracts, intact Dpp is in abundance, although a smear of apparent Dpp degradation products is evident.(42) Human Dpp is in higher abundance in growing root tips than in mature dentin,(46) suggesting that Dpp is degraded. MEP1B is the first enzyme shown to degrade Dpp, and this capability suggests that it may play a role in Dpp degradation when the free calcium concentration is low.

The predentin matrix of developing pig teeth was immunostained by a commercial antibody raised against rhBMP-1, and an immunopositive band comigrating with rhBMP-1 was isolated from porcine dentin powder and cleaved Dspp-FRET at the G–D bond. Originally, we interpreted these findings to mean that BMP-1 is normally expressed and secreted by odontoblasts and is likely to be the enzyme that specifically hydrolyzes Dspp to release Dpp in vivo. In addition, *Bmp1* mRNA was readily amplified from cells (mainly odontoblasts) that adhere to dentin following removal of the pulp. However, similar RT-PCR reactions for other members of the astacin family were positive, and a Western blot analysis using the commercial BMP-1 antibody demonstrated its cross-reactivity with other astacin proteases. The current evidence implicates astacin proteases in the cleavage of Dspp to generate Dpp and rules out hydrolysis of Dspp by MMP-2, MMP-8, MMP-9, MMP-20, Mt1-MMP, Mt3-MMP, Klk4, or Dpp. All three of the 7 astacin proteases studied here cleaved the Dpp bond that releases Dpp, so it seems likely that this might be a property of all members of this group.

BMP-1 was originally reported to be the enzyme that removes the C-terminal propeptides from collagen, but later studies of knockout mice revealed that multiple astacins process collagen, so there is molecular redundancy for that function. We suspect a similar redundancy in the processing of Dspp to yield Dpp. Further studies are required that characterize which astacins are expressed by odontoblasts, whether or not they are able to catalyze the Dpp cleavage, and whether the single and multiple conditional knockouts of the genes encoding these enzymes block the processing of Dspp in vivo.
