# Supplementary material for: Astacin Proteases Cleave Dentin Sialophosphoprotein (Dspp) to Generate Dentin Phosphoprotein (Dpp)
Source: J Bone Miner Res. 2010 Aug 4;26(1):220–8. doi: 10.1002/jbmr.202 (PMC3179315; doi:10.1002/jbmr.202)
Supplement: Supplementary file 1 [file jbmr0026-0220-SD1.doc]

**Astacin Proteases Cleave Dentin Sialophosphoprotein (Dspp)**

**to Generate Dentin Phosphoprotein (Dpp)**

Shuhei Tsuchiya^1,2^, James P. Simmer^1^, Jan C-C. Hu^1^,

Amelia S. Richardson^1^, Fumiko Yamakoshi^1^, and Yasuo Yamakoshi^1^

1 Department of Biologic and Materials Sciences, University of Michigan School of Dentistry, Ann Arbor, MI 48108

2 Nagoya University Graduate School of Medicine 65 Tsurumai Showa-ku, Nagoya 466-8550, Japan

**List of Figures Provided in the Appendix**

1) **Suppl. Fig. 1. Activities of proteases.** This figure shows gelatin and casein zymograms of each protease used in the study, the digestion of control FRET peptides by enzymes that don’t show up on the zymograms, and the stability of Dspp-FRET when incubated with Dpp.

2) **Suppl. Fig. 2. Cleavage of Dspp-FRET.** This figure shows the results of the digestion of Dspp-FRET (Abz-YEFDGKSMQGDDPN-KDnp) by 11 proteases: porcine Mmp-20, rhMT1-MMP, rhMT3-MMP, rhMMP-2, rhMMP-8, rhMMP-9, porcine Klk4, human placental plasmin, rhBMP1, rhMEP1A and rhMEP1B.

3) **Suppl. Fig. 3. Cleavage of mutDspp-FRET.** This figure shows the results of the digestion of mutDspp-FRET (Abz-YEFDGKSIEGDDPN-KDnp) by 11 proteases: porcine Mmp-20, rhMT1-MMP, rhMT3-MMP, rhMMP-2, rhMMP-8, rhMMP-9, porcine Klk4, human placental plasmin, rhBMP1, rhMEP1A and rhMEP1B.

4) **Suppl. Fig. 4. Dentin powder fractions assayed for enzymes that cleave Dspp-FRET.** This figure shows the results of digesting Dspp-FRET with the eight dentin powder extracts: G1S, G1P, A, AN, TN, G2S, G2P, or R. The G1S, G1P, and the TN extracts cleaved Dspp-FRET. The activity in G1S and G1P could be attributed to Klk4. The TN extract was further fractionated and characterized.

5) **Suppl. Fig. 5. Identifying a BMP1-like protein in the TN extract.** This figure shows the fractionation of the TN extract by anion exchange chromatography and characterization of the collected samples by SDS-PAGE and Western blot analyses. Samples 45 and 46 was immunopositive using the antibody raised against rhBMP1.

| 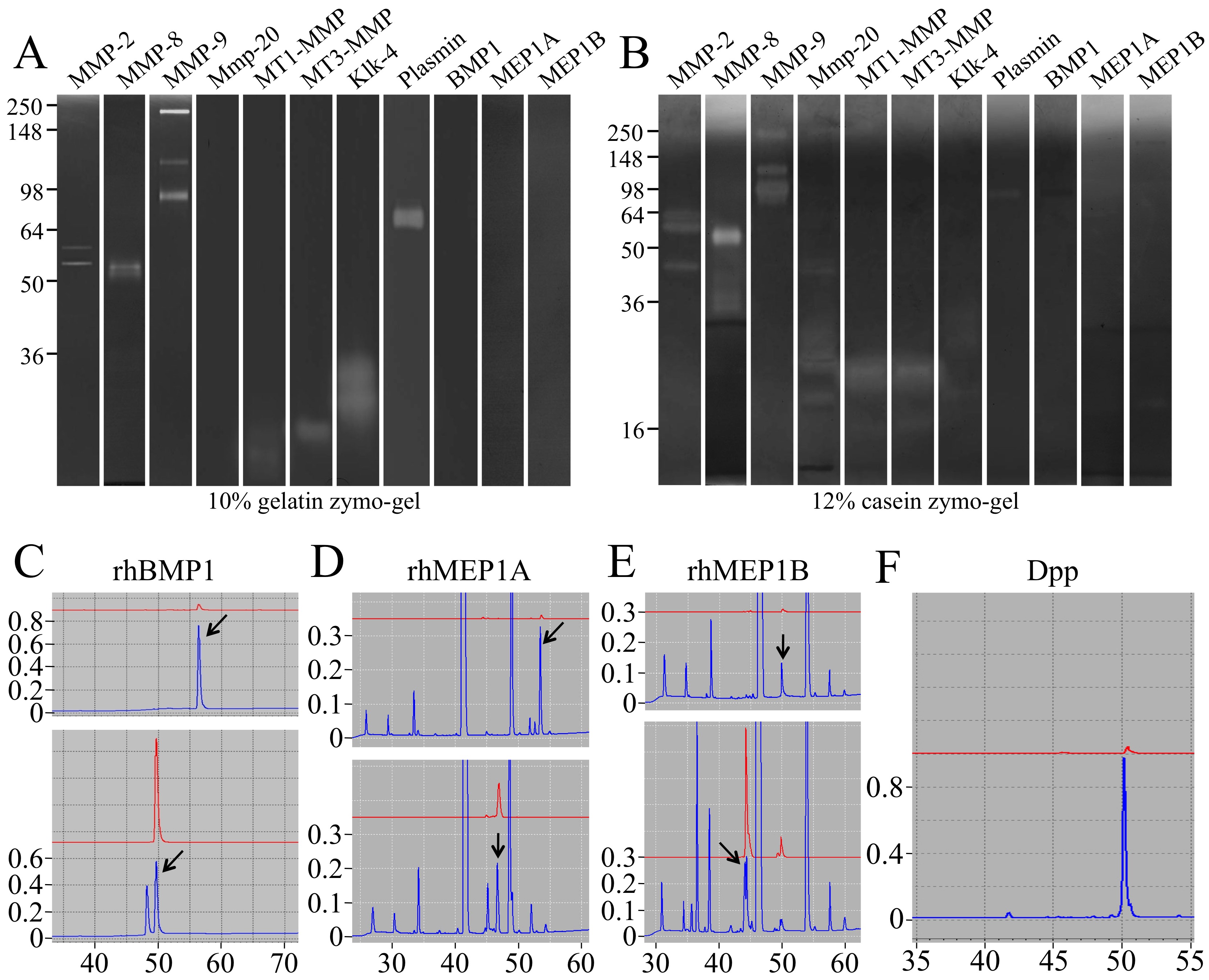 |
| --- |
| **Suppl. Fig. 1. Activities of proteases.** ***A:*** 10% gelatin zymogram and ***B:*** 12% casein zymogram showing commercially available recombinant human proteases MMP-2, MMP-8, MMP-9, MT1-MMP, MT3-MMP, BMP1, MEP1A, MEP1B and placental plasmin, and porcine Mmp-20 and Klk4 isolated from developing teeth. ***C-F:*** C-18 RP-HPLC chromatograms of FRET peptides containing appropriate target sequences to test for enzyme activity. Cleavage of the peptide is determined by appearance of a fluorescent peak at a different retention time than the uncleaved peptide. ***C top:*** chromatogram of uncleaved FRET peptide Mca-YVADAPK(Dnp)-OH (arrow); ***C bottom:*** chromatogram of same peptide cleaved by rhBMP1 (arrow). ***D top:*** Chromatogram of uncleaved target peptide Mca-YVADAPK(Dnp)-OH (arrow) mixed with the serine protease inhibitor AEBSF (that generates multiple background peaks); chromatogram of same peptide cleaved by activated rhMEP1A with the serine protease inhibitor AEBSF. ***E top:*** Chromatogram of uncleaved target peptide Mca-SEVNLDAEFRK(Dnp)RR-NH2 (arrow) mixed with the serine protease inhibitor AEBSF; ***E bottom:*** chromatogram of same peptide cleaved by activated rhMEP1B with the serine protease inhibitor AEBSF (arrow). ***F:*** Dspp-FRET peptide (Abz-YEFDGKSM QGDDPN-KDnp) after 48 h incubation with porcine 100 µg of Dpp showing no activity. |

| 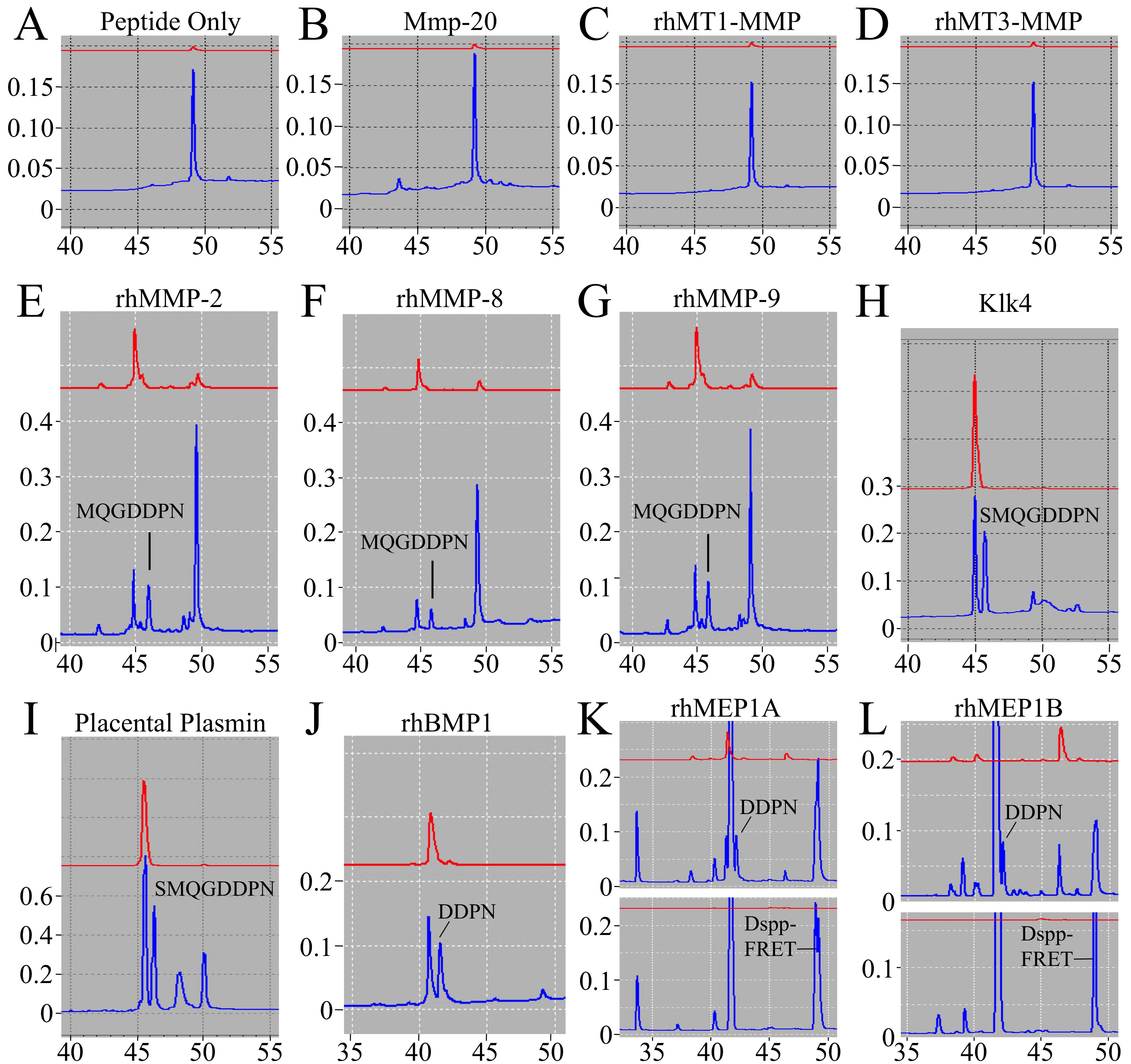 |
| --- |
| **Suppl. Fig. 2. Cleavage of Dspp-FRET.** C-18 RP-HPLC chromatograms of Dspp-FRET showing absorbance at 220 nm (blue) and fluorescence (red) after incubation with proteolytic enzymes. ***A:*** the uncleaved Dspp-FRET peptide (Abz-YEFDGKSMQGDDPN-KDnp). Peaks showing strong fluorescence contain the N-terminal Abz label but not the C-terminal Dnp. ***B-L:*** Dspp-FRET following incubation with ***B:*** porcine Mmp-20; ***C:*** rhMT1-MMP; ***D:*** rhMT3-MMP; ***E:*** rhMMP-2, ***F:*** rhMMP-8, ***G:*** rhMMP-9, ***H:*** porcine Klk4, ***I:*** human placental plasmin, ***J:*** rhBMP1, ***K:*** rhMEP1A and ***L:*** rhMEP1B. Klk4, plasmin, rhBMP1, rhMEP1A and rhMEP1B were able to cleave Dspp-FRET. Mass spec analyses identified the cleavage sites, which were confirmed by Edman sequencing of the labeled peaks. Klk4 and plasmin cleaved the peptide between lysine and serine generating the peptide SMQGDDPN. The rhBMP1, rhMEP1A, and rhMEP1B cleaved the peptide between glycine and aspartic acid generating the DDPN peptide. |

| 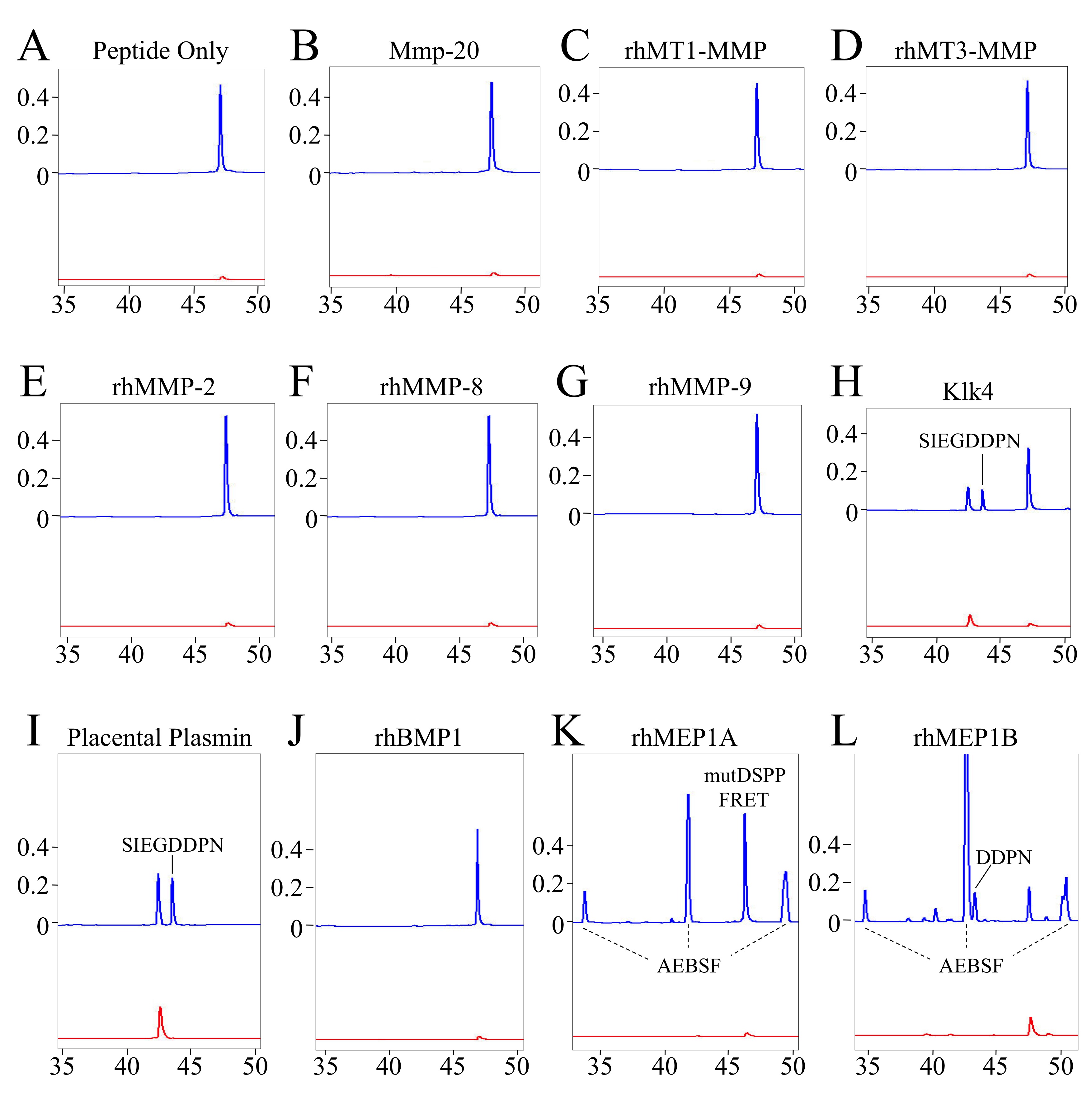 |
| --- |
| **Suppl. Fig. 3. Cleavage of mutDspp-FRET.** C-18 RP-HPLC chromatograms of mutDspp-FRET showing absorbance at 220 nm (blue) and fluorescence (red). ***A:*** the uncleaved mutDspp-FRET peptide (Abz-YEFDGKSIEGDDPN-KDnp). Peaks showing strong fluorescence contain the N-terminal Abz label, but not the C-terminal Dnp. ***B-L:*** mutDspp-FRET following incubation with each protease. ***B:*** porcine Mmp-20; ***C:*** rhMT1-MMP; ***D:*** rhMT3-MMP; ***E:*** rhMMP-2; ***F:*** rhMMP-8; ***G:*** rhMMP-9; ***H:*** porcine Klk4; ***I:*** human placental plasmin; ***J:*** rhBMP1; ***K:*** rhMEP1A; and ***L:*** rhMEP1B. Note that rhMMP-2, rhMMP-8, and rhMMP-9 did not cleave mutDspp-FRET even though they could cleaved Dspp-FRET but not at the G-D bond. The rhMEP1B was able to cleave both Dspp- and mutDspp-FRET at the G-D bond. These results suggest that failure to cleave a mutated sequence cleavage may not prove that an enzyme cleaved the true Dpp cleavage site in the wild-type sequence. |

| 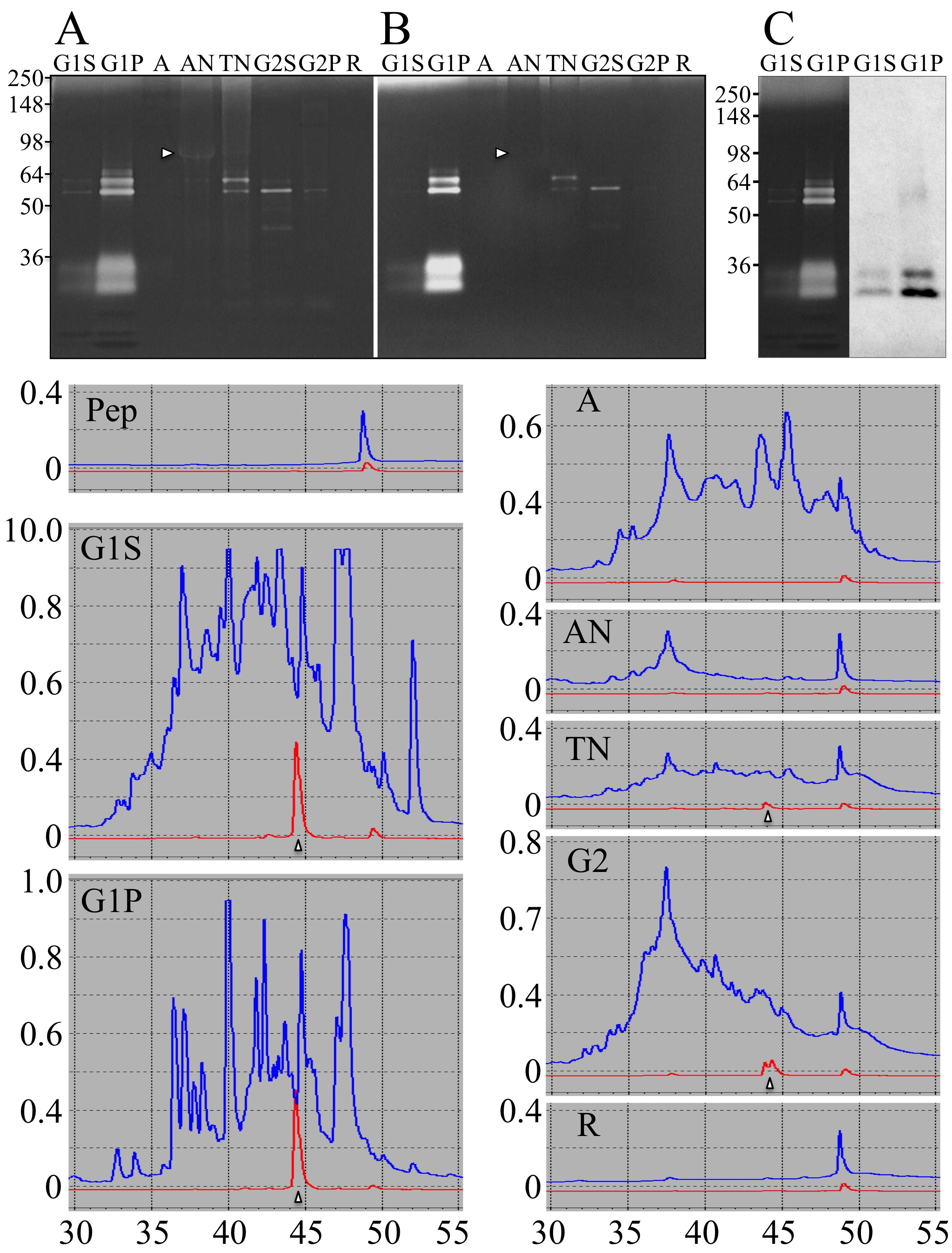 |
| --- |
| **Suppl. Fig. 4. Dentin powder fractions assayed for enzymes that cleave Dspp-FRET.** ***A:*** 10% gelatin zymogram (CBB stained) showing the 8 primary extracts from porcine dentin powder. Note that Dpp in the AN extract and does not stain with CBB, so it shows up as a negative band (arrowhead). ***B:*** same gel as in ***A*** stained with CBB plus Stains-All. Dpp stains with stains all and disappears (arrowhead). ***C:*** 10% gelatin zymogram and Western blot showing that Klk4, which digests Dspp-FRET, is the strong doublet in the first guanidine fractions (G1S and G1P). **Bottom:** Digestions of Dspp-FRET with dentin powder fractions. C-18 RP-HPLC chromatograms showing absorbance at 220 nm (blue) and fluorescence (red) of the DSPP-FRET peptide incubated for 20 h alone (Pep), or with the G1S (270 µg), G1P (270 µg), A (260 µg), AN (280 µg), TN (250 µg), G2 (G2S+G2P, 270 µg), or R (230 µg) dentin fractions. The TN fraction showed weak, but specific activity and was chosen for further characterization. Arrowheads mark fluorescent peaks caused by cleavage of the peptide. |

| 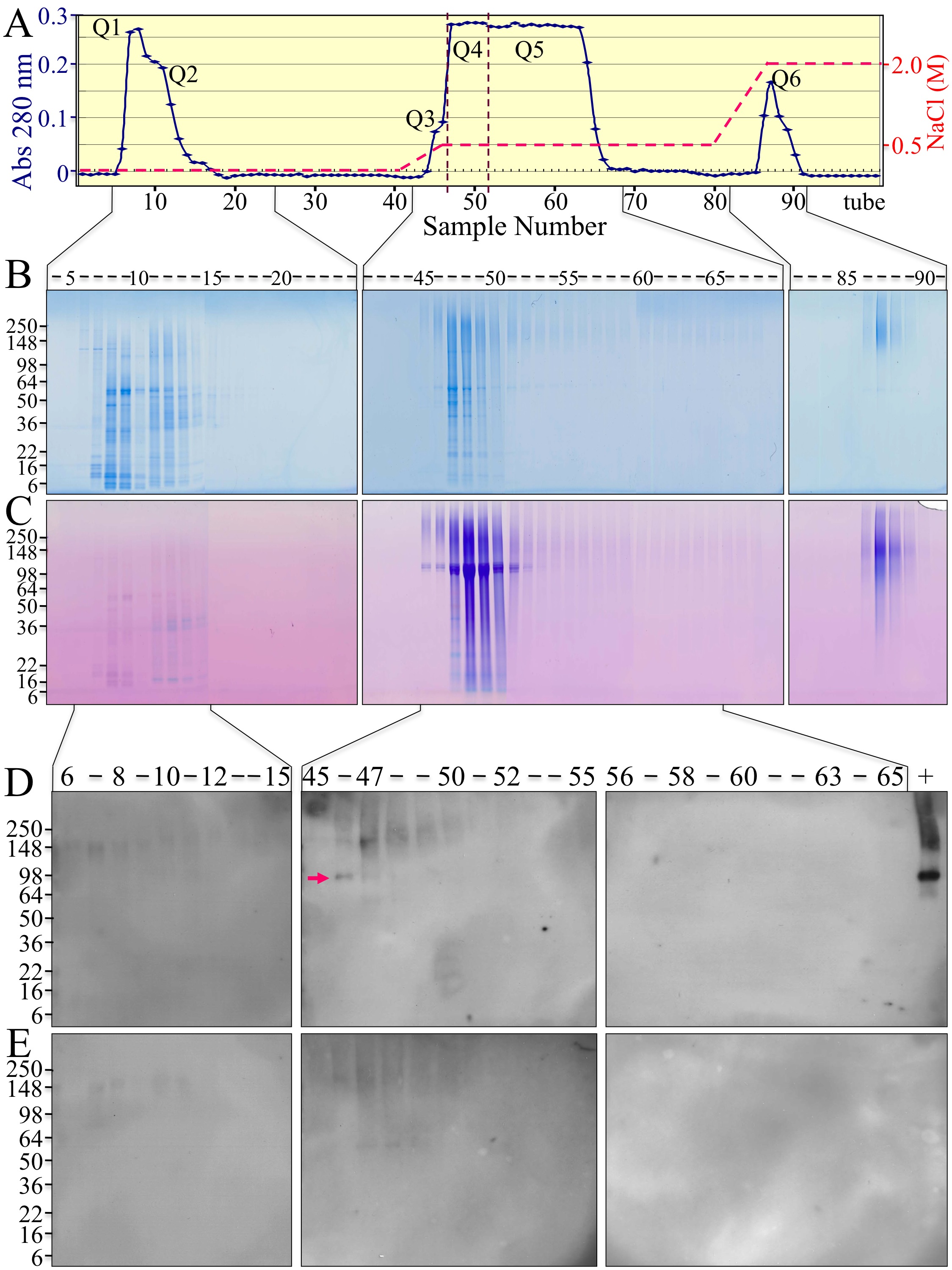 |
| --- |
| **Suppl. Fig. 5. Identifying a BMP1-like protein in the TN extract.** ***A:*** Chromatogram of the TN extract separated by anion exchange chromatography. Samples were collected every 20 min, with 100 samples collected. Most of the protein concentrated in six chromatographic peaks (Q1 to Q6). ***B:*** SDS-PAGE stained with CBB. ***C:*** SDS-PAGE stained with Stains-All showing the protein contents of the samples collected from the six anion exchange peaks. Lane numbers correspond to samples from the anion exchange chromatography. ***D:*** Western blot using the BMP1 antibody as a probe and rhBMP1 (+) as a positive control/size indicator. An immunopositive band was identified in samples 45 and 46 (red arrow) ***E*.** Western blot negative control omitting the primary (BMP1) antibody. The secondary antibody alone tends to stain immunoglobulins in the fractions. Note the Bmp1 positive band in ***D*** is not stained in ***E***. |
